# Silencing of the GluN1-NMDA Glutamate Receptor Subunit by Intranasal siRNA Increases the Latency Time for Seizures in the Pilocarpine Rodent Model of Epilepsy

**DOI:** 10.3390/ph15121470

**Published:** 2022-11-26

**Authors:** Raphaela Gonçalves Barros Perri, Anieli Gaverio Mantello, Daiane Santos Rosa, Renê Oliveira Beleboni

**Affiliations:** 1 Department of Biotechnology, University of Ribeirão Preto, Ribeirão Preto 14096-300, SP, Brazil; 2 School of Medicine, University of Ribeirão Preto, Ribeirão Preto 14096-300, SP, Brazil

**Keywords:** antiepileptic agents, temporal lobe epilepsy (TLE), glutamatergic neurotransmission, pilocarpine model, interference RNA, intranasal route

## Abstract

Temporal lobe epilepsy (TLE) is the most prevalent and treatment-refractory type of epilepsy. Among the different mechanisms associated with epileptogenesis, overstimulation of glutamatergic neurotransmission has been associated with the onset and progression of seizures in TLE. Experimental evidence indicates that blocking the N-methyl-D-aspartate (NMDA) receptor or suppressing the expression of its subunit, mainly GluN1, may be effective in preventing epileptic seizures. Small interfering RNA (siRNA) has received attention as a potential therapeutic tool due to the inhibition of gene expression in some diseases. The present work evaluated the potential silencing effect of intranasal administration of an siRNA conjugate against the GluN1 subunit in animals submitted to the pilocarpine model of epilepsy. The results showed that the siRNA conjugate transfection system silences the GluN1 subunit in the hippocampus of rats when administered intranasally. As demonstrated by the RT-qPCR and Western blotting approaches, the silencing of GluN1 was specific for this subunit without affecting the amount of mRNA for other subunits. Silencing increased the latency time for the first tonic–clonic seizure when compared to controls. The overlapping of findings and the validation of the intranasal route as a pharmacological route of siRNA targeting the GluN1 subunit give the work a significant biotechnological interest.

## 1. Introduction

Epilepsy affects about 65 million people around the world; it is part of a neurological group of diseases characterized by the manifestation of recurrent and self-sustaining epileptic seizures [1]. Temporal lobe epilepsy (TLE) is the most common form among the different types of epilepsy and is also the most refractory to conventional pharmacological treatments [2,3]. Status epilepticus (SE) is a marked event assembling with the onset, maintenance, and progress of TLE. As a representation of one of the main pathophysiological characteristics of TLE, and closely related to SE, different structural changes are observed in the hippocampal neural networks in animal models and in human patients, including hippocampal sclerosis, reactive gliosis, mossy fiber sprouting, aberrant synaptic reorganizations, and granular cell dispersion [4].

Glutamatergic neurotransmission has an important role in TLE, especially when considering NMDA-type glutamatergic receptors (N-methyl-D-Aspartate). In fact, a significant increase in the expression and associated function of NMDA receptors occurs in the hippocampus, mainly in the later stages of SE. This event, when associated with the increased GABAergic inhibitory function losses, implies the establishment and maintenance of SE, progressing sequentially to the self-sustaining seizures characteristic of TLE [5,6,7]. The main functional components of the NMDA receptor involve different subunits: the determinant/fundamental GluN1 subunit and the complementary ones ranging most commonly from GluN2A-D types [8,9,10,11]. The receptor-associated channel becomes activated when glycine or D-serine binds to its GluN1 binding sites, and L-glutamate binds to its site in the complementary GluN2B subunit. The different GluN2B subunits are also often sites for different allosteric modulators, and those subunits are the ones that give the biggest functional diversity to NMDA receptors [8,9,10]. An increase in the hippocampal GluN1 subunit expression has been demonstrated for animals submitted to the pilocarpine epilepsy model, in hippocampal cell cultures, and in tissues surgically removed from epileptic patients, reinforcing its role in TLE [12]. Given the importance of the NMDA receptor in epileptogenesis as well as in the maintenance and progression of seizures in TLE, it is expected to have an attractive relevance as a potential target for different therapeutic approaches.

The effective treatment for TLE is challenging and complex. Most antiepileptic drugs prescribed for seizure control are ineffective and can cause serious side effects. Most frequently, clinicians choose the strategy of polytherapy as one means of over-treatment in epilepsy, using a combination of different antiepileptic drugs (AEDs) that show in general different modes of actions, a practice which increases the therapeutic efficacy in many cases. However, this also increases the incidence and severity of side effects, limiting the effectiveness of the drug combination and resulting in a suboptimal risk-to-benefit assessment that negatively affects the therapeutic adherence of patients [13]. The search for new strategies applied to the treatment of TLE is urgently required due to the high incidence and economic impact caused by this disease, which is associated with huge personal and social losses for patients [14]. In this rationale, the use of animal models is still necessary for the development of new AEDs. The pilocarpine-induced epilepsy model reproduces the main pathophysiological and behavioral changes observed in the human TLE [15]. This animal model has been widely applied to a better morphofunctional characterization of the cellular and molecular bases involved in epileptogenesis as well as in prospecting for new AEDs useful in restoring the optimal balance between inhibitory and excitatory neurotransmission lost along TLE [16].

More study of the RNA interference method and its correlated delivery systems as well as exploration of alternative administration routes could be helpful to understand and potentially treat different disorders, including TLE. This technique consists of a post-transcriptional gene-silencing mechanism mediated by small double-stranded RNAs (dsRNAs) designed to identify a target mRNA sequence and mediate its neutralization, thus modulating the expression of a corresponding protein [17]. Efficient delivery, structural stability and specificity in the cellular environment are the biggest technical obstacles to the practical use of RNAi. However, several well-engineered delivery systems and the use of alternative administration routes have shown promising results for its experimental or clinical use [18]. Given the NMDA receptor function gain in TLE, this work aims to evaluate the potential silencing effect of a specific siRNA-nanoparticle system directed to the GluN1-NMDA subunit. This siRNA-nanoparticle system was administered by the intranasal route in animals submitted to the pilocarpine epilepsy model, and the resulting potential antiepileptic performance of this experimental approach was investigated.

## 2. Results

### 2.1. RT-qPCR Analysis: siRNA Intranasal Injection Silences the GluN1 Gene Selectively in the Hippocampus

**Figure 1:**
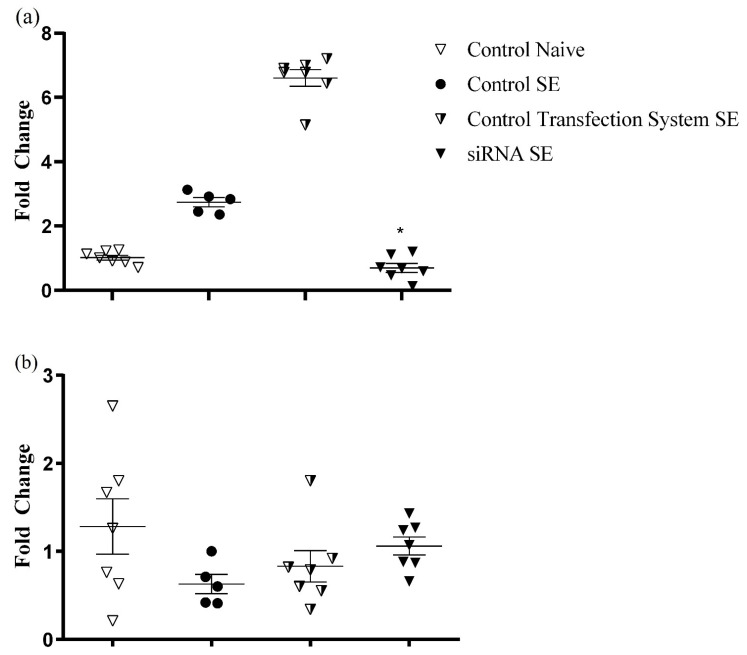
Relative gene expression of the GluN1 gene normalized with GAPDH (5 μg/rat siRNA-GluN1), compared to controls. Bars show the fold change (2^−ΔΔCt^) average for each group. Statistical analysis was performed using one-way ANOVA followed by Dunnett’s multiple comparison test and Shapiro–Wilk * (*p* < 0.05). Animal groups were divided as follows: CONTROL NAÏVE (no intranasal treatment/no SE induction after 14 days of group formation); CONTROL SE (no intranasal treatment/SE induction after 14 days of group formation); CONTROL TRANSFECTION SYSTEM SE (intranasal treatment with transfection system/SE induction after 14 days of intranasal treatment); siRNA SE: (intranasal treatment with siRNA/SE induction after 14 days of intranasal treatment). (**a**) Hippocampus; (**b**) Cortex.

On the 14th day after animal group arrangements (CONTROL NAÏVE and CONTROL SE) or intranasal treatments (CONTROL TRANSFECTION SYSTEM SE and siRNA SE), the rats were submitted to the pilocarpine model for SE induction (except for the CONTROL NAÏVE group), and 24 h later, they were sacrificed, and the hippocampus and cortices of each animal were collected. Most importantly, the group treated with GluN1-siRNA/transfection system (siRNA SE) showed a significant reduction in this subunit expression compared to the control group (CONTROL SE) (0.45-fold change, *p* < 0.05) in the hippocampus RT-qPCR analysis [F (3, 22) = 262.5, *p* < 0.0001] (Figure 1a). In the cortex, no differences in RT-qPCR analysis were observed between the experimental and control groups [F (3, 22) = 1.745, *p* = 0.1872] (Figure 1b). 

### 2.2. RT-qPCR Analysis: NR1 Subunit Silencing by siRNA Intranasal Injection Does Not Affect mRNA-GluN2A/2B Gene Expression

**Figure 2:**
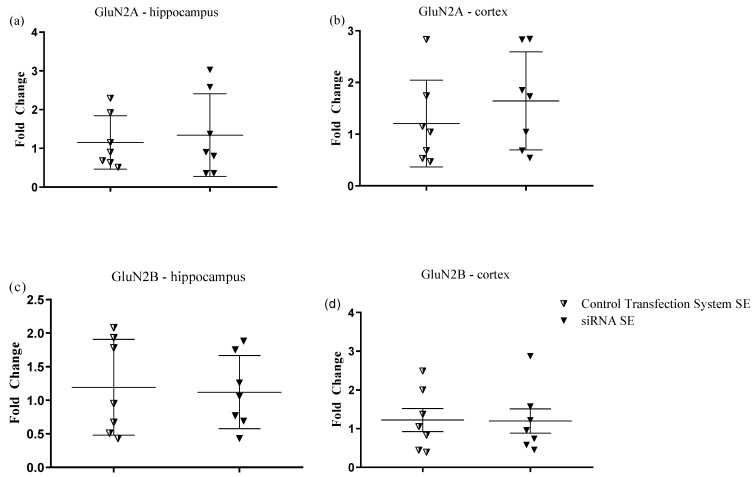
Relative gene expression of the GluN2A/2B gene normalized with GAPDH, compared to controls. Bars show the fold change (2^−ΔΔCt^) average for each group. Statistical analysis was performed using two-way ANOVA, *t*-test, and Shapiro–Wilk test. Animal groups were divided as follows: CONTROL TRANSFECTION SYSTEM SE (intranasal treatment with transfection system/SE induction after 14 days of intranasal treatment) and siRNA SE: (intranasal treatment with siRNA/SE induction after 14 days of intranasal treatment). (**a**) GluN2A—Hippocampus; (**b**) GluN2A—Hippocampus; (**c**) GluN2B—Hippocampus; (**d**) GluN2B—Cortex.

To complement the gene expression analysis, samples of the hippocampus and cortex from animals submitted to NR1 subunit silencing were also analyzed for the mRNA GluN2A and GluN2B subunits expression by using RT-qPCR. Analysis showed no difference between CONTROL TRANSFECTION SYSTEM SE and siRNA SE 14 after intranasal treatments of animals and 24 h later SE. To attain a higher experimental interest, only the animal groups CONTROL TRANSFECTION SYSTEM SE and siRNA SE were studied in this set of experiments. The results show that treatment with siRNA-GluN1 does not affect GluN2A and GluN2B genes expression in the hippocampus [F (6, 6) = 1.708, *p* = 0.2659] (Figure 2a), [F (6, 6) = 1.208, *p* = 0.4122] (Figure 2b) and in the cortex [F (6, 6) = 1.977, *p* = 0.2137] (Figure 2c), [F (6, 6) = 1631 *p* = 0.2836] (Figure 2d).

### 2.3. Western Blotting Analysis: SiRNA Intranasal Injection Reduces GluN1 Protein Levels in the Hippocampus

**Figure 3:**
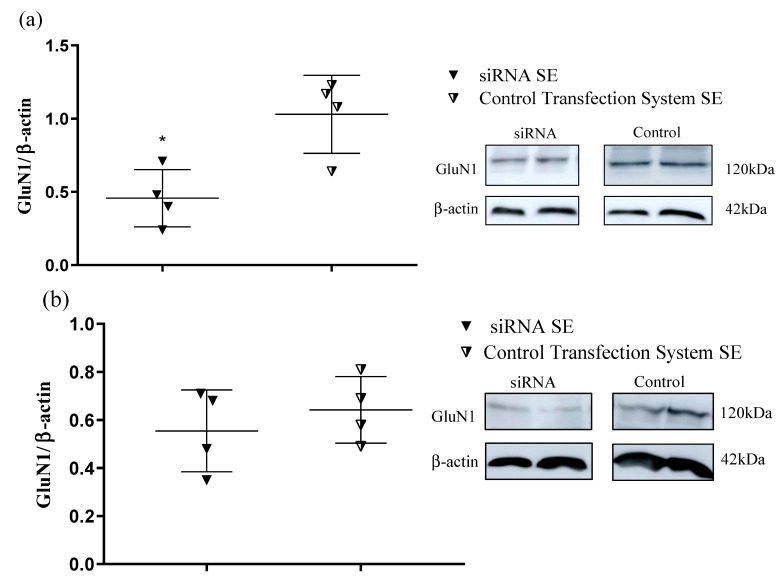
GluN1 protein expression (14 days after intranasal treatment and 24 h later SE) normalized with β-actin, relative to control. Statistical analysis was performed using two-way ANOVA, *t*-test, and Shapiro–Wilk test * *p* < 0.05. To optimize the experimental interest, animal groups were divided as follows: CONTROL TRANSFECTION SYSTEM SE (intranasal treatment with transfection system/SE induction after 14 days of intranasal treatment) and siRNA SE: (intranasal treatment with siRNA/SE induction after 14 days of intranasal treatment). (**a**) GluN1—Hippocampus; (**b**) GluN1—Cortex.

To investigate whether SiRNA intranasal injection reduces GluN1 protein levels in the hippocampus and cortex of animals, Western blotting analysis was employed to compare CONTROL TRANSFECTION SYSTEM SE and siRNA SE animal groups. Western blotting analysis showed that silencing the GluN1 subunit not only reduced the mRNA expression level (RT-qPCR) but also effectively reduced the protein (GluN1) level expressed in the hippocampus of animals treated with siRNA. The decrease in the protein expression level was around 51% when CONTROL TRANSFECTION SYSTEM SE and siRNA SE animal groups were compared 14 h after intranasal treatment and 24 h later SE [F (1, 6) = 83.52, *p* < 0.0001] (Figure 3a). In contrast, there was no significant difference in the cortex between these animal groups [F (1, 6) = 2.316, *p* < 0.1788] (Figure 3b).

### 2.4. Anti-Epileptic Performance: GluN1 Silencing in the Hippocampus Increases Latency Time for the First Seizure in Treated Animals

**Figure 4:**
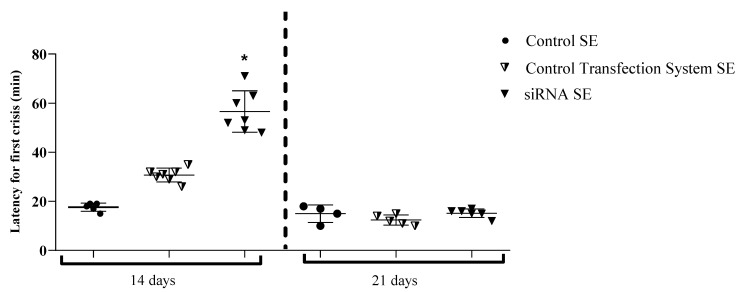
Latency for first tonic–clonic seizures in animals submitted to SE in a pilocarpine-induced epilepsy model. The bars represent the mean ± standard deviation. * *p* < 0.05 compared to the 14-day control SE group, *p* < 0.05 compared to the 21-day control SE group. Groups 14 days and 21 days were individually analyzed by the one-way ANOVA test and Shapiro–Wilk. Animal groups were divided as follows: CONTROL SE (no intranasal treatment/SE induction after 14 days of group formation); CONTROL TRANSFECTION SYSTEM SE (intranasal treatment with transfection system/SE induction after 14 days of intranasal treatment) and siRNA SE: (intranasal treatment with siRNA/SE induction after 14 days of intranasal treatment). Only for analysis of antiepileptic performance experiments and for a better exploration of results, an additional animal group was SE-induced 21 days after the group arrangement (CONTROL SE) or intranasal treatments (CONTROL TRANSFECTION SYSTEM SE and siRNA SE groups) and was evaluated separately from the other animal groups in which SE was induced after 14 days of their treatment/formation.

The criteria used to assess the antiepileptic performance of the treatment were as follows: seizure severity and latency to the first epileptic seizure within three hours of SE, 14 or 21 days after treatment with the siRNA-GluN1/transfection system. The treatment with the siRNA-GluN1 subunit significantly increased latency for the first seizures (14 days) compared to control groups. In fact, when analyzing the latency period for the onset of tonic–clonic seizures, animals treated with siRNA-GluN1 increased the latency compared to Control SE in 14 days [F (2, 16) = 79.92, *p* = 0.0001]. The experimental and control groups 21 days after treatment showed no statistical differences regarding the latency time for the first seizure [F (2, 12) = 2.088, *p* = 0.1667] (Figure 4). 

## 3. Discussion

The results presented here show for the first time that the selected transfection system grouped with the designed siRNA selectively silences the GluN1 subunit in the hippocampus of SE-induced animals when intranasally administrated without significantly affecting the cortex. This finding was demonstrated by both the RT-qPCR (mRNA expression) and Western blot (protein density level) experimental approaches, showing that these data corroborate each other.

The selective GluN1 subunit silencing in the hippocampus is important to TLE since this brain region centralizes most of the cellular lesions, neurochemical changes resulting from status epilepticus (SE), and, therefore, the onset, progress, and maintenance of epileptogenesis. In fact, SE in TLE has been demonstrated in both animal models and human patients, showing similar well-characterized lesions involving neuronal death in the dentate gyrus, in the horn of Amon CA1-CA3, and significant hippocampal reactive gliosis [19,20]. Regarding the most important neurochemical changes during the onset of SE, there is a decrease in the GABA-mediated inhibitory neurotransmission through its GABAA receptors caused by the hypofunction and/or decrease in density of this receptor. Conversely, in the later stages of SE, there is an increase in the expression and/or associated function of the glutamatergic NMDA receptor, which in turn, when associated with more losses of GABAergic inhibitory functions, results in the establishment and maintenance of SE that progresses sequentially to the self-sustaining seizures characteristic of TLE [5,6,7]. In this regard, Wasterlain et al. (2013) demonstrated the relocation of GluN1-NMDA receptor subunits from the cytoplasm to the surface of neurons in the dentate gyrus of the hippocampus in animal models of lithium/pilocarpine and SE after the injection of neurokinin B, producing an increase in functional NMDA receptor density and a consequent increase in neuronal firing in this brain region.

It is important to note that NMDA receptors exert important physiological roles in the central nervous system (CNS), altering, maturing, and potentiating synaptic structures through the neuronal plasticity modulation crucially involved in memory and learning processes, especially in the cortex and subcortical regions, including the hippocampus [5]. Thus, the silencing of the NMDA receptor in brain areas in which lesions resulting from SE are most concentrated (such as the hippocampus) proves to be potentially advantageous, considering preliminary aspects of pharmacological safety and tolerability for new potential probe drugs. However, other brain areas need to be further investigated in addition to the cortex and hippocampus to have a more comprehensive conclusion about the hippocampal selectivity presented by the siRNA studied here. In this case and considering the different physiological roles of NMDA receptors in different brain areas, pharmacological safety tests must be extended, and potential changes in the cognitive capacity of the GluN1-SiRNA-treated animals must be investigated from different toxicological perspectives for a more comprehensive conclusion about this potential advantage. 

The GluN1 subunit was specifically silenced by the used siRNA sequence without significant alterations in the amount of mRNA of other subunits (GluN2A and GluN2B) as assessed by RT-qPCR. As discussed in this work, many studies show the beneficial effect of GluN1 blockade on epilepsy, either by pharmacological antagonists or by genetic manipulation (transgenic approaches, knockout, or use of RNAi) [21,22,23]. In the adult hippocampus, NMDA receptors are usually made from the essential GluN1 subunit, which is important for receptor functioning. The NMDA receptor is composed of a GluN1 subunit combined with a GluN2 and/or GluN3. In fact, besides the crucial role for functional NMDA receptors, quantitative hybridizations analysis has shown that the GluN1 is the most expressed subunit in different areas of the CNS when compared to the other subunits. The GluN1 subunit begins to be expressed from the fourteenth day of embryonic development, reaching an expression peak after the third week of the rodent birth, then barely changing until adulthood [24]. Studies involving the overexpression or suppression effects in this subunit have opened new avenues for understanding its action both in physiological and pathological brain conditions. Obviously, several mechanisms need to be unraveled regarding the exact role of NMDA receptors in epilepsy, which may make possible therapies that are more effective and safer than those currently applied [25].

Since glutamate and its NMDA receptor are involved in different types of epilepsy, pharmacological inhibition of this receptor or even gene expression suppression, particularly the GluN1 subunit, may offer protection against epileptic seizures, which reinforces the role of this receptor in epileptogenesis as well as in the potential treatment of TLE [11,26]. The selective hippocampal suppression of GluN1 induced by the intranasal siRNA and its validation for both RT-qPCR and Western Blotting analysis in this work seems to be correlated to the increased latency for epileptic seizure onset of animals treated with this intranasal SiRNA when submitted to the pilocarpine/SE model and compared to control group(s) as evidenced in the antiepileptic performance test. Similar data to this work were presented by Chapman et al. (1996), in which the increase in seizure latency was the most promising result observed in animals submitted to audiogenic crises after using an antisense oligodeoxynucleotide directed against GluN1-NMDA.

In this work, a decrease in the frequency and/or severity of seizures was not observed. However, the data presented above for increasing seizure latency time were robust enough to indicate some antiepileptic efficiency to siRNA intranasally administered although further studies are still necessary. Additional experiments, some of them currently in course, would include the study of new doses of siRNA and different severities or times of SE as well as more comprehensive studies of seizures in animals treated with siRNA by video-electroencephalography for a better investigation of this potential SiRNA antiepileptic activity. Briefly, NMDA receptor suppression as shown here may protect hippocampal neurons against death. This may partially explain the siRNA antiepileptic activity presented in this study. In fact, neuronal loss induced in the pilocarpine model occurs through different mechanisms [23]. Specifically, the excessive increase in intracellular Ca++ resulting from cholinergic activation and the generation of free radicals as intermediate products, which in turn can potentiate the excessive release of glutamate and related inflammatory mediators [27]. Preliminary data from our laboratory, through Nissl staining, showed that CA1, CA3, and dentate gyrus (HGD) hippocampus regions presented a significantly higher number of viable cells for animals treated with siRNA when compared to the control group that also passed through SE. This may mean that siRNA treatment protects neurons from SE-induced death in these hippocampal regions, which can be interpreted as a neuroprotective effect (unpublished data).

## 4. Materials and Methods

### 4.1. Drugs and Reagents

Ketamine (Ketalar^®^-Parke Davis Warner Lambert), xylazine (Hertape Calier), sodium thiopental (Tiopentax^®^-Cristália), High-Capacity cDNA Reverse Transcription Kit (Thermo Fisher, Waltham, MA, USA) were used. Pilocarpine hydrochloride, SYBR Green Jumpstart-S448, TRI reagent, RNAse-free water, methyl scopolamine bromide, protease inhibitor cocktail, Bradford reagent and siRNA-GluN1 sequence were purchased from Sigma-Aldrich, St Louis, MO, USA. The other materials were as follows: transfection system–5031 (Altogen Biosystems, Las Vegas, NV, USA), ECL molecular weight standard, nitrocellulose membrane, ECL chemiluminescence reagent (GE Healthcare Life Sciences, Piscataway, New Jersey, USA), NMDAR1 primary antibody–NB300-118 (R&D system, USA), Antibody b-actin clone c4–MAB1501 (Merck Millipore, Temecula, CA, USA), Secondary antibody IgG-BP-HRP-SC516102 (Santa Cruz Biotechnology, Dallas, TX, USA).

### 4.2. Animals

Wistar male rats (7–8 weeks, 180–220 g) were used in this study. The animals were kept in our animal facility under standard acclimatization conditions, with free access to water and food (12 h light/dark cycle; light on at 7 am and light off at 7 pm) with the room temperature at 20 ± 2 °C. All experimental procedures were approved by the Ethics Committee of the University of Ribeirão Preto (protocol number: 10/2015). The experimental protocols involving animals followed the Brazilian College of Animal Experimentation rules and the American Guidelines for Animal Care. The animal treatments and the experiments were carried out by different researchers in a blinding protocol, and the animals were randomly distributed between the groups.

The animals were divided into the following groups (*n* = 5–7): (1) Naive group (CONTROL NAIVE): animals that did not receive any intranasal treatment and were not submitted to the SE induction protocol/pilocarpine model; (2) untreated and SE-induced group (CONTROL SE): animals that did not receive any type of intranasal treatment but were submitted to the SE induction protocol/pilocarpine model; (3) group treated with transfection system and induced by SE (CONTROL TRANSFECTION SYSTEM SE): animals that received the transfection system with 5% glucose added to the saline solution by the intranasal route and which were induced by the SE/pilocarpine model; (4) group treated with siRNA/transfection system and induced of SE (siRNA SE): animals treated with siRNA (5 µg/animal by intranasal route) conjugated to the transfection system and submitted to the SE induction protocol/pilocarpine model.

The SE induction procedure is described below. SE was induced after 14 days of each treatment or animal group formation/arrangement when appropriate. The SiRNA dosage (5 µg/animal) was based on the pilot-scale testing as well as the descriptions written by Tan et al. (2005); the waiting time for the SE induction and the procedure for intranasal administering of each material of interest (described below) were also defined from previous scale-based experiments.

### 4.3. SiRNA-GluN1: Preparation and Intranasal Administration

The siRNA sequence toward GluN1-coding mRNA was synthesized by Sigma-Aldrich (USA) (Forward, 5′ GACAAGUUCAUCUACGCAA [dT][dT] 3′, Reverse, 5′ UUGCGUAGAUGAACUUGUC [dT][dT] 3′) and selected according to the highest score for gene expression modulation and according to in silico validation by that manufacturer. 

The lyophilized RNA oligonucleotide (at 100 μM in RNAse-free water) was conjugated to an in vivo transfection system kit (NANOPARTICLE-cat # 5031 from Altogen Biosystems, Las Vegas, Nevada, USA) according to the manufacturer’s guidance. The transfection system was assembled from positive nanoparticles containing coordinated iron ions. Briefly, siRNA and transfection reagent were mixed and remained at rest for 15 min at room temperature. Next, the enhancer (reaction intensifier) was added, and the solution was stirred in a vortex, followed by standing for five minutes at room temperature. The final volume was completed with a 5% glucose solution.

Intranasal administration was performed on 10% ketamine hydrochloride (10 mg/kg, i.p) and xylazine (8 mg/kg, i.p) anesthetized rats. Following Rodriguez et al. (2017) [28], with modifications, these animals were then placed in the supine position and received repeated volumes of 5 μL administered alternately in each nostril of the animal using a micropipette, at 2–3-min intervals, until the final volume of 50 μL of siRNA or 5% solution + transfection system was achieved in each animal belonging to the different groups (CONTROL TRANSFECTION SYSTEM SE or siRNA SE). This allowed the animals treated with siRNA + transfection system to reach a 5 μg dose of siRNA/rat (Tan et al., 2005). 

### 4.4. SE Induction/Pilocarpine-Induced Epilepsy Model and Analysis of Intranasal siRNA Antiepileptic Performance Evaluation

Fourteen days after the intranasal route treatments or animal group formation when the case, rats from CONTROL SE, CONTROL TRANSFECTION SYSTEM SE, and siRNA SE groups were induced to SE receiving pilocarpine (320 mg/kg) via i.p., while animals from CONTROL NAIVE group received an equivalent volume of 0.9% saline (i.p.). To avoid peripheral effects of pilocarpine, animals submitted to SE were pre-treated with methyl scopolamine bromide dissolved in sterile saline (1 mg/kg) 30 min before i.p. pilocarpine administration [29]. Three hours after pilocarpine administration, the rats were treated with sodium thiopental (30 mg/kg i.p.) to interrupt seizures and decrease the animal mortality rate.

The classification scale for limbic seizures [29]. was used for SE definition/identification as well as for crisis-severity scoring. During the three-hour SE induction, the rats were kept in individual acrylic boxes for behavioral observation. The latency for the onset of spontaneous seizures and the severity of seizures were analyzed/recorded based on animal behavioral evaluation in a comparison between control and experimental groups (antiepileptic performance). Importantly, and only for analysis of antiepileptic performance, additional animal groups (*n* = 5–7/group) were SE-induced 21 days after the group formation or intranasal treatments and were evaluated separately from the other animal groups in which SE was induced after 14 days of their treatment/formation.

Twenty-four hours after SE onset, in the animal groups in which SE was induced after 14 days of their treatment/formation, the hippocampus and cortices were removed from each animal for RT-qPCR and Western blotting experiments (described below). The structures from both brain hemispheres were quickly dissected into a Petri dish fixed on ice, washed with cold sterile saline, weighed, and stored at −80 °C.

### 4.5. Real-Time PCR

RNA was isolated and purified from individual samples from the hippocampus and cortex using TRI Reagent (Sigma-Aldrich, St Louis, MO, USA) according to the manufacturer’s instructions. RNA-extracted concentration from samples was determined by UV spectrophotometry (260 nm), and its integrity was verified by electrophoresis in 1% denaturing agarose gel under constant-voltage conditions (80 V) for 60 min. The first-strand cDNA was synthesized using the High-Capacity cDNA Reverse Transcription kit (ThermoFisher™) in a total volume of 20 μL containing 1 μg of the total RNA. Real-time PCR was performed in a final volume of 25 μL containing 1 μL of the cDNA (SYBR^®^ Green JumpStart Taq ReadyMix for RT-qPCR kit # S4438-Sigma Aldrich-USA). The amplification reactions were in triplicate and taken to the thermal cycler MXPRO 3005 (Stratagene™) with the following cycling conditions: 94 ºC for 2 min, 40 cycles at 94 °C for 15 s, and 60 °C for 1 min. The following sets of primers were used: GluN1-Fwd.5′-GCA AGA ATG AGT CAG CCC AC-3′, Rev. 5′-CAG TCA CTC CGT CCG CAT AC-3′ (Tan et al., 2005). The RNAm-GluN2A/GluN2B gene expression experiment was analyzed in animals subjected to NR1 subunit silencing; the primers used were as follows: GluN2A–Fwd. 5′ TCCACTCAAGGAATCCTTGTGAGA-3′ and (GluN2B–Fwd. 5′-CUCAGAAGAAGAAUCGGAA-3′, Rev. 5′-UUCCGAUUCUUCUUCUGAG-3′) [30], GADPH was used as a reference gene (GAPDH-Fwd. 5′-TGC ACC AAC TGC TTAG-3′, Rev. 5′-GGA TGC AGG GAT GTTC-3′) [31]. 

The RT-qPCR data were analyzed according to the 2^−ΔΔct^ method proposed by Livak and Schmittgen (2001) [32]. The calculation was made in relation to the expression of the CONTROL SE group, based on the comparison of the Ct values, expressed in arbitrary units, between the groups of samples at the precise moment when the PCR reached the exponential amplification phase. According to this model, the following equation was applied:

fold-change = 2^−ΔΔct^

ΔCt = Target gene Ct − Reference gene Ct

ΔΔCt = ΔCt1 − ΔCt2

ΔCt1 = sample of interest

ΔCt2 = control group sample

### 4.6. Western Blotting

For the purpose of experimental optimization, and according to a higher experimental interest, in the Western blot experiments, only the animal groups CONTROL TRANSFECTION SYSTEM SE and siRNA SE were compared. Hippocampal and cortical tissues were separately homogenized in the RIPA buffer (0.2 mg/µL) containing 1% protease inhibitor (Sigma-Aldrich). Protein concentration was determined using the Bradford method [33]. Samples (4 μg) were mixed with Laemmli buffer containing 0.125 M Tris (pH 6.8), 20% Glycerol, 10% β-mercaptoethanol, 4% SDS, and 0.002% Bromophenol blue and heated at 70 °C for 5 min. The protein content was loaded onto a polyacrylamide gel (4–10% Bis-Tris) and separated by electrophoresis using a Bio-Rad system with protein molecular weight standards (Rainbow-GE) at 80 V for 2.5 h. The proteins were then transferred to a 45 µm nitrocellulose membrane (GE Healthcare Life science) at 100 V for 1 h. The membranes were washed with 0.1 M Tris–Tween 20, blocked with 0.1 M Tris added of 5% nonfat dry milk and then incubated with the primary antibody (NMDAR1 NB300-118–R&D system: 1:1000) at 4 °C overnight. Afterward, the membranes were rinsed and incubated with the corresponding secondary antibody (anti-mouse IgG-BP-HRP–SC-516102–Santa Cruz Biotechnology: dilution 1:1000) for 1 h at room temperature. After washing them again with 0.1 M Tris-Tween20, the membranes were ready for the blocking phase and subsequent staining with the internal reaction control anti-β actin antibody (Anti-Actin Antibody, clone c4–cat. # MAB1501–Merck Millipore: dilution 1:100,000). After washing, the bands were detected by chemiluminescence using the ECL reagent (Amersham ECl Prime WB detection reagent–GE^®^ Healthcare Life Sciences). The ECL reagent excess was removed, followed by reading the membrane (ImageQuant LAS 500). The band intensity was quantified using the ImageJ software. In the supplementary material, the figures of the membranes (Figures S1 and S2) and tables with the data of the antibodies used are presented (Table S1). 

### 4.7. Statistical Analysis

The data from the RT-qPCR experiments were analyzed using one-way ANOVA followed by Dunnett’s multiple comparison test and two-way ANOVA. Western blot data were analyzed using the non-parametric *t*-test and two-way ANOVA. Antiepileptic performance was evaluated by the one-way ANOVA test. The Shapiro–Wilk test was also performed for all experiments. Statistical analyses and graphic constructions were performed using the GraphPad Prism 7.0 program (USA), and the value of *p* < 0.05 was considered statistically significant.

## 5. Conclusions

Indeed, the NMDA receptor is not the only explanation for epileptogenesis in TLE, in SE development, or even the single target in the treatment of such a complex pathology. However, it is possible to assume that the selected transfection system used in this work was effective in protecting GluN1-siRNA from enzymatic degradation, effective in its solubilization/delivery along with the nasal mucus and in overcoming biological barriers such as the arachnoid barrier when considering the SiRNA crossing from the nasal cavity directly to the CNS. Although new experiments are still necessary, such data are pioneering for GluN1 subunit silencing and bring interesting biotechnological perspectives, especially when considering the GluN1-SiRNA hippocampal selective delivery and the GluN1-siRNA/transfection system effectiveness as discussed above. In fact, one of the technical difficulties of TLE treatment using the RNAi technique is the need to reduce NMDA receptor expression in a sustained manner over time and/or chronically [34]. In this context, the intranasal route has great value because it is easily accessible, well tolerated by patients, relatively safe, and easy to use continuously. The results presented in this work validate the intranasal pathway as useful in silencing or modulating the hippocampal NMDA receptor with associated antiepileptic activity, which is desirable considering its gain in function in TLE and the functional role of its GluN1 subunit.

## Data Availability

Data is contained within the article and supplementary materials.

## Supplementary Materials

The following supporting information can be downloaded at: https://www.mdpi.com/article/10.3390/ph15121470/s1, Figure S1: GluN1 protein expression (14 days after intranasal treatment and 24 h later SE) normalized with β-actin, relative to control—Hippocampus; Figure S2: GluN1 protein expression (14 days after intranasal treatment and 24 h later SE) normalized with β-actin, relative to control—Cortex; Table S1: Used antibodies.
