# Supplementary material for: Silencing of the GluN1-NMDA Glutamate Receptor Subunit by Intranasal siRNA Increases the Latency Time for Seizures in the Pilocarpine Rodent Model of Epilepsy
Source: Pharmaceuticals (Basel). 2022 Nov 26;15(12):1470. doi: 10.3390/ph15121470 (PMC9785971; doi:10.3390/ph15121470)
Supplement: Supplementary file 1 [file pharmaceuticals-15-01470-s001.zip › pharmaceuticals-1946155-supplementary.pdf]

Supplementary Material

Silencing of the GluN1-NMDA Glutamate Receptor Subunit by Intranasal siRNA Increases the Latency Time for Seizures in the Pilocarpine Rodent Model of Epilepsy

Raphaela Gonçalves Barros Perri [1], Anieli Gaverio Mantello [1], Daiane Santos Rosa [1] and Renê Oliveira Beleboni [1,2,] *

[1] Department of Biotechnology, University of Ribeirão Preto, Ribeirão Preto 14096-300, SP, Brazil
[2] School of Medicine, University of Ribeirão Preto, Ribeirão Preto 14096-300, SP, Brazil
* Correspondence: rbeleboni@unaerp.br; Tel.: +55-16-3603-6827

SUPPLEMENTARY IMAGES - WESTERN BLOT

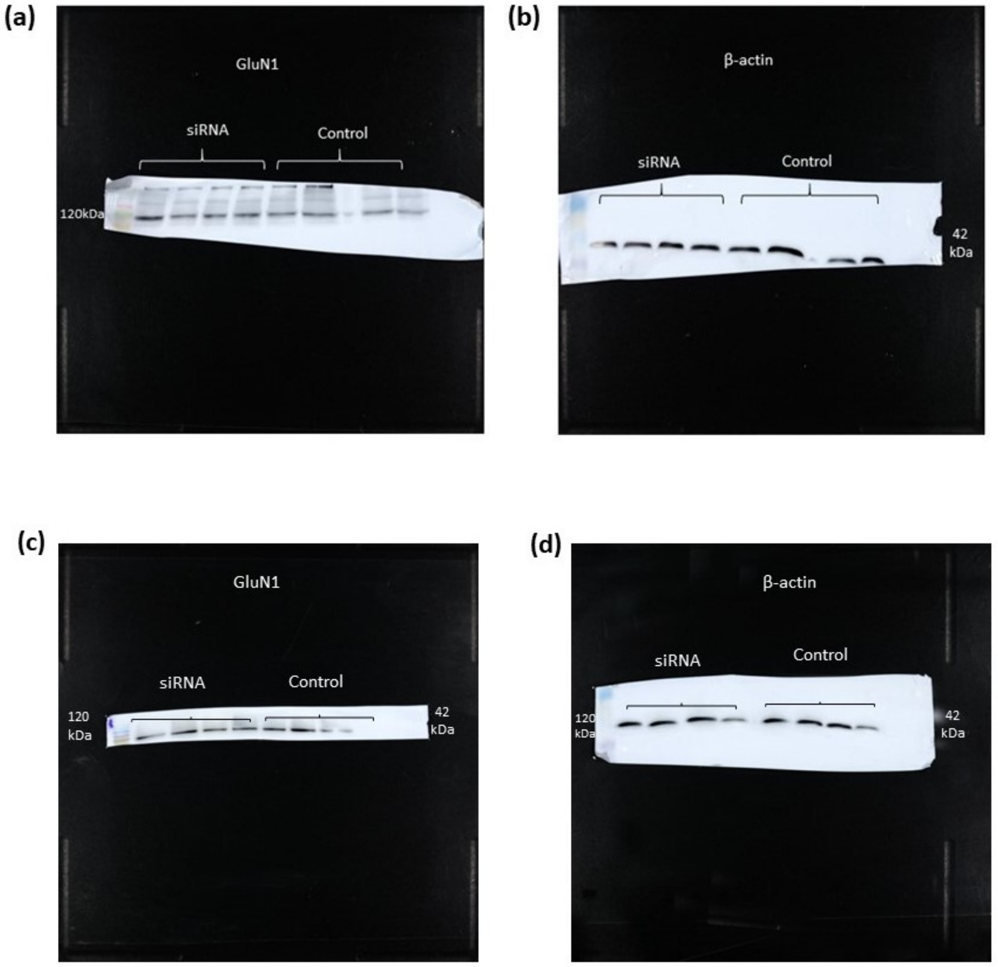

Figure S1. GluN1 protein expression (14 days after intranasal treatment and 24 h later SE) normalized with β-actin, relative to control, (**a**) GluN1 – Hippocampus (membrane 1); (**b**) β -actin – Hippocampus (membrane 1); (**c**) GluN1 – Hippocampus (membrane 2); (**d**) β -actin – Hippocampus (membrane 2).

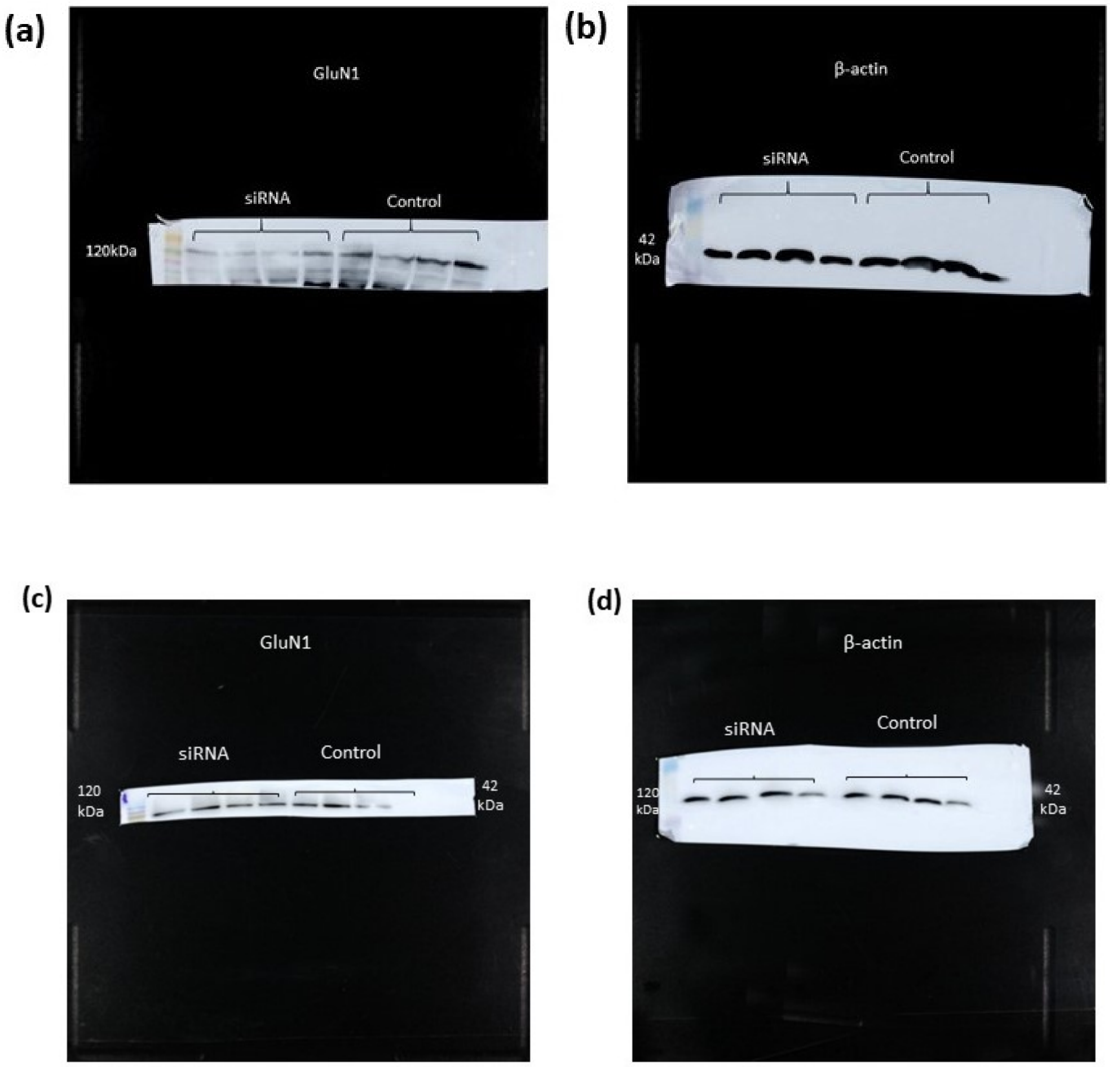

Figure S2. GluN1 protein expression (14 days after intranasal treatment and 24 h later SE) normalized with β-actin, relative to control, (**a**) GluN1 – Cortex (membrane 1); (**b**) β -actin – Cortex (membrane 1); (**c**) GluN1 – Cortex (membrane 2); (**d**) β -actin – Cortex (membrane 2).

NOTE: Full images related to hippocampal and cortex samples for GluN1 protein (NMDA glutamatergic receptor subunit), with all protein bands and markers. Membranes were cut in half to optimize the antibody incubation and washing process.

SiRNA-GluN1

As stated in the Manuscript: The siRNA sequence toward GluN1-coding mRNA was synthesized by Sigma-Aldrich (USA) (*Forward, 5′ GACAAGUUCAUCUACGCAA [dT][dT] 3′, Reverse, 5′ UUGCGUAGAUGAACUUGUC [dT][dT] 3′*) and selected according to the highest modulation score of gene expression and according to the manufacturers in silico validation. Consult the data sheet provided by the company Sigma-Aldrich (one of the leading global companies in the provision and design of siRNAs).

Datasheet: http://www.mdpi.com/authors (accessed on 1 January 2021)).

ANTIBODIES INFORMATION

Table S1. Used antibodies.

Antibody	Target	Host and Clonality	Supplier	Catalog Number	Dilution
					WB
IgG-BP-HRP Antibody secondary	m-IgGκ BP	Mouse Monoclonal	Santa Cruz Biotechnology	SC-516102	1:1000
NMDAR1 Antibody primary	GluN1	Mouse Monoclonal	R&D system	NB300-118	1:1000
Anti-actin	Actin	Mouse - C4, monoclonal	Merck Millipore	# MAB1501	1:100000

- Anti-Actin, clone C4, Monoclonal Antibody (MAB1501)

Certificate of Analysis: http://www.mdpi.com/authors (accessed on 22 January 2021)).

- NMDAR1 Antibody (R1JHL) NB300-118

Datasheet: http://www.mdpi.com/authors (accessed on 23 January 2021)).

STATISTICAL DATA OF THE ANALYSIS PERFORMED

- **FIGURE 1a**

ANOVA table	SS	DF	MS	F (DFn, DFd)	P value
Treatment (between columns)	154,4	3	51,47	F (3, 22) = 262,5	P<0,0001

Shapiro-Wilk test				
W	0,9526	0,9241	0,7633	0,9521
P value	0,7530	0,5566	0,0174	0,7492
Passed normality test (alpha=0.05)?	Yes	Yes	No	Yes
P value summary	ns	ns	*	ns

- **FIGURE 1b**

ANOVA table	SS	DF	MS	F (DFn, DFd)	P value
Treatment (between columns)	1,460	3	0,4868	F (3, 22) = 1,745	P=0,1872

Shapiro-Wilk test				
W	0,9700	0,9023	0,8385	0,9612
P value	0,8984	0,4229	0,0963	0,8290
Passed normality test (alpha=0.05)?	Yes	Yes	Yes	Yes
P value summary	ns	ns	ns	ns

- **FIGURE 2a**

ANOVA table	SS	DF	MS	F (DFn, DFd)	P value
Row Factor	6,090	6	1,015	F (6, 6) = 1,708	P=0,2659
Column Factor	0,1245	1	0,1245	F (1, 6) = 0,2094	P=0,6634

Shapiro-Wilk test		
W	0,8589	0,8591
P value	0,1481	0,1487
Passed normality test (alpha=0.05)?	Yes	Yes
P value summary	ns	ns

- **FIGURE 2b**

ANOVA table	SS	DF	MS	F (DFn, DFd)	P value
Row Factor	5,268	6	0,8779	F (6, 6) = 1,208	P=0,4122
Column Factor	0,6732	1	0,6732	F (1, 6) = 0,9263	P=0,3730

Shapiro-Wilk test		
W	0,8955	0,8569
P value	0,3045	0,1419
Passed normality test (alpha=0.05)?	Yes	Yes
P value summary	ns	ns

- **FIGURE 2c**

ANOVA table	SS	DF	MS	F (DFn, DFd)	P value
Row Factor	3,212	6	0,5354	F (6, 6) = 1,977	P=0,2137
Column Factor	0,01858	1	0,01858	F (1, 6) = 0,06861	P=0,8021

Shapiro-Wilk test		
W	0,9399	0,8536
P value	0,6374	0,1326
Passed normality test (alpha=0.05)?	Yes	Yes
P value summary	ns	ns

- **FIGURE 2d**

ANOVA table	SS	DF	MS	F (DFn, DFd)	P value
Row Factor	4,875	6	0,8125	F (6, 6) = 1,631	P=0,2836
Column Factor	0,002314	1	0,002314	F (1, 6) = 0,004646	P=0,9479

Shapiro-Wilk test		
W	0,8493	0,9270
P value	0,1211	0,5253
Passed normality test (alpha=0.05)?	Yes	Yes
P value summary	ns	ns

- **FIGURE 3a**

ANOVA table	SS	DF	MS	F (DFn, DFd)	P value
Column Factor	1,389	1	1,389	F (1, 6) = 83,52	P<0,0001

Unpaired t test	
P value	<0,0001
P value summary	****
Significantly different (P < 0.05)?	Yes
One- or two-tailed P value?	Two-tailed
t, df	t=7,044, df=12

Shapiro-Wilk test		
W	0,9740	0,8569
P value	0,9257	0,1420
Passed normality test (alpha=0.05)?	Yes	Yes
P value summary	ns	ns

- **FIGURE 3b**

ANOVA table	SS	DF	MS	F (DFn, DFd)	P value
Column Factor	0,1189	1	0,1189	F (1, 6) = 2,316	P=0,1788

Unpaired t test	
P value	0,1353
P value summary	ns
Significantly different (P < 0.05)?	No
One- or two-tailed P value?	Two-tailed
t, df	t=1,601, df=12

Shapiro-Wilk test		
W	0,8972	0,9601
P value	0,3146	0,8198
Passed normality test (alpha=0.05)?	Yes	Yes

P value summary	ns	ns

- **FIGURE 4**

Shapiro-Wilk test						
W	0,8810	0,9119	0,9747	0,8947	0,8659	0,9524
P value	0,3140	0,4090	0,9303	0,4051	0,2102	0,7540
Passed normality test (alpha=0.05)?	Yes	Yes	Yes	Yes	Yes	Yes
P value summary	ns	ns	ns	ns	ns	ns

14 days

ANOVA table	SS	DF	MS	F (DFn, DFd)	P value
Treatment (between columns)	4839	2	2419	F (2, 16) = 79,92	P<0,0001

21 days

ANOVA table	SS	DF	MS	F (DFn, DFd)	P value
Treatment (between columns)	24,37	2	12,18	F (2, 12) = 2,088	P=0,1667
